# Between a rock and a hard place: balancing roles of clinician and co-researcher in a primary health care setting - A qualitative study

**DOI:** 10.1186/s12998-026-00681-9

**Published:** 2026-10-01

**Authors:** Anna Allen-Unhammer, Nina Køpke Vøllestad, Anne Marit Mengshoel

**Affiliations:** Department of Public Health and Interdisciplinary Health Sciences, Institute of Health and Society, Faculty of Medicine, University of Oslo, Postbox 1130 Forskningsparken, Oslo, 0318 Norway

**Keywords:** Primary health care, Private practitioners, Role conflicts, Recruitment, Qualitative interviews, Focus group interviews

## Abstract

**Background:**

The participation of clinicians in clinical research is necessary, but balancing research and clinical duties can be challenging. We explored chiropractors’ experiences of participating in a clinical trial on spinal manipulation of the neck in primary health care (PHC).

**Methods:**

A qualitative design was adopted, underpinned by social interactionism. Five virtual focus group interviews were performed between August-November 2023, including 15 chiropractors, who were involved in a clinical trial in PHC. Thematic analysis was applied.

**Results:**

The interviewees were motivated to participate in the clinical trial to contribute to professional development through investigating the use of spinal manipulation. However, patient recruitment and adherence to the treatment protocol were more difficult than anticipated, especially when patients realised that the treatment would be determined by randomisation and because the chiropractors had to deliver the sham/placebo interventions. The interviewed chiropractors continually negotiated conflicting obligations of clinicians, co-researchers, and breadwinners, shifting loyalties between these roles in response to situational demands.

**Conclusions:**

The clinicians’ motivations to take part in the trial varied but were in general rooted in a wish to contribute to strengthening the chiropractic profession. The negotiation of multiple roles and shifting loyalties rendered them moral agents. Our findings illustrate the social and relational nature of participating as a co-researcher in a trial conducted in a chiropractic PHC context. Our study also highlights a need for better support systems for research in PHC. This calls for political and organisational will to establish systems that provide necessary support to researchers and clinical co-researchers.

**Supplementary Information:**

The online version contains supplementary material available at https://doi.org/10.1186/s12998-026-00681-9.

## Background

The collaboration between researchers and clinicians is central when conducting clinical research in hospitals and primary health care (PHC). Indeed, the interprofessional interplay between researcher and clinician is important to guide the successful execution of trial recruitment [1]. Clinicians typically screen and recruit patients meeting the inclusion criteria, and deliver study interventions alongside routine patient care, adopting roles of clinician and co-researcher [2–4]. Such multiple roles may produce ethical and practical dilemmas for clinicians because clinical practice emphasises individualised care, while clinical trial protocols require clinicians to follow standardised procedures [5, 6]. Further, clinicians’ personal beliefs and expectations can shape judgments about who they deem eligible to take part in clinical studies [1, 2, 7, 8]. Eligible patients may not be invited, leading to selection bias that can undermine a study’s validity [1, 2, 9–11]. Thus, adopting the role of co-researcher can present an array of challenges for clinicians.

There is extensive literature on factors that facilitate or impede recruitment to clinical studies [8, 12–15]. These are typically classified into contextual-, practitioner- and participant-related factors [1, 9, 14, 16]. Contextual factors include reimbursement and research support systems for recruitment [14, 17]. An additional factor is the availability of eligible patients, as it is commonly found that the number of participants available for recruitment is much lower than initially anticipated [8, 16, 18–21]. Practitioner-related factors include clinicians finding study information to be insufficient, resulting in clinicians not fully understanding what is required of them [9, 22]. This can impact on how well they inform potential study participants about clinical studies [8, 23]. A clinician’s relationship with patients can also affect their willingness to recruit patients [14, 18, 24], highlighting the importance of the therapeutic alliance in clinical practice [8, 25]. Patient-related factors include treatment preferences [18, 26]; despite patients receiving detailed study information, they may decline to participate because of a concern for not getting their desired treatment [8, 15]. Some studies suggest that several factors may be at play simultaneously [3, 8]. In the current study, we want to explore how these elements interact in a PHC context, using a chiropractic trial as a case.

Three Cochrane reviews examine studies of recruitment in a variety of contexts [12–14], but they do not differentiate between hospital and PHC contexts. A systematic review of recruitment in general practice points out that contextual differences are likely to limit the transferability of findings between contexts [15]. This is for several of the following reasons. Large hospitals, for instance university hospitals, have the infrastructure and routines for undertaking research and actively involve clinicians in the research process, encouraging more ownership to clinical studies [17]. In contrast, there is seldom research support and less of a tradition to participate in clinical studies in a PHC setting [27, 28]. Thus, for PHC clinicians, participation is driven by personal motivation rather than institutional requirements [27]. Undertaking research in PHC can affect income because clinicians may not get reimbursed for their efforts [29]. In addition, long-term patient-clinician relationships in PHC may increase perceived loyalty. This may be less challenging in hospitals, where care is typically team-based and episodic. In both settings, recruitment is time consuming, and high workloads tend to hinder recruitment [15, 30].

In this study, the objective was to examine chiropractors’ experiences and their reflections about participating in a multicenter clinical trial in a Norwegian PHC context.

## Methods

### Context

In Norway, chiropractors are licensed PHC clinicians who operate as private practitioners and are partly integrated into the public health system. They can certify sick leave for musculoskeletal conditions [31] and refer for specialist care and diagnostic imaging [32], akin to physicians and manual therapists. Chiropractic care costs are mainly covered by patients, with a small reimbursement from the health authorities.

Chiropractors involved in the clinical trial (Clinicaltrials.gov identifier: NCT05374057) were recruited by the investigators of the trial, through their clinical network and through information at conferences. They agreed to screen, enroll and randomise eligible patients with acute neck pain (NP) to spinal manipulation, sham manipulation, ibuprofen, or placebo medicine. These chiropractors delivered the allocated treatments without reimbursement, and the patients were asked to avoid other therapies during the 14-day intervention period before seeking additional care, for further details see the protocol [33].

### Study design and theoretical framework

Our study adopts a descriptive qualitative design to capture nuances of experiences among co-researchers participating in the same trial, and data were generated using focus group interviews [34]. The interviewing and analysis of this qualitative study are underpinned by a theoretical understanding of social interactionism. The basic assumption of social interactionism is that the way an individual acts is rooted in the meaning a situation has for that person. This meaning is continuously created and reshaped through everyday interactions with others and various contexts [35, 36]. This assisted us in understanding how the chiropractors actively interpreted and negotiated their actions as co-researchers, through their own personal values, professional knowledge and experiences [35, 37]. In other words, experiences were understood as both personal and unique and were at the same time developed and shaped through social interactions. Thus, the chiropractors could make sense of and influence each other’s experiences through discussions and reflections in the focus groups. During the interviews and analysis, the researchers aimed for interpretation and reflexivity rather than striving for neutrality [38].

The female moderator of the sessions was an experienced, qualitative researcher (AMM) with a physiotherapy and manual therapy background, who was unfamiliar to the chiropractors prior to study commencement and was not involved in the trial. This allowed for open, naïve questioning. The other researcher (AAU) with a chiropractic background, was familiar with the participating chiropractors, through her previous role as a monitor of the clinical trial. It was decided beforehand that AAU would not lead the interviews but be present to assist the moderator. The interviews were conducted virtually using Zoom to include clinicians nationwide.

### Research ethics

Ethical approval was granted by the Data Protection Officer at Akershus University Hospital (ref. 21/13390). The clinicians received an email explaining the purpose of the study, procedures, and data handling. Verbal and written informed consent, including permission for audio-video recording was obtained. Participation was voluntary, and withdrawal was permitted prior to article submission. After the interviews, video files were deleted and the audio files transferred to the University of Oslo’s secure research server (TSD). The transcripts were anonymised, and the reporting procedures prioritised confidentiality, given the small number of chiropractors in Norway.

### Study participants and focus group interviews

All 43 chiropractors active in the trial until June 2023 were invited by telephone; 15 (35%) agreed, and none dropped out. Thus, we adopted a convenience sampling method. Nonetheless, the participants were a heterogeneous group varying in age, gender, education, geographical region, nationality and clinical experience. Reasons for non-participation were not collected. To promote diverse discussions and gather clinicians with varied experiences as co-researchers, we intentionally constructed the focus groups to include clinicians who had recruited many, few, and no trial participants.

Five focus groups were performed between August-November 2023. The number of participants in each focus group varied (three focus groups had three participants, one focus group had two participants, and one focus group had four participants). The small numbers in the groups were mainly due to the chiropractors’ hectic time schedules. The interview guide (Table 1) was prepared based on researchers’ prior meetings with the clinicians and qualitative literature [8, 9]. The moderator introduced herself, described her research interests briefly and the main reasons for doing the study. Open questions were asked to elicit experiences and opinions [39], and data from early interviews informed subsequent interviews [40]. The interviews began with general themes and proceeded to specific prompts as the discussions evolved. The interviews lasted between 60 and 90 min including a 10-minute break.

**Table 1:** Interview guide for focus group interviews with thematically organised questions

The moderator’s focus group interview guide was used to initiate the interviews. Broad questioning on areas such as clinical setting, location, job role was used to initiate the focus group discussions, followed by questions relating to factors/conditions that might affect recruitment.
Icebreaker: Welcome and presentation
**1. Contextual factors**:
Reasons for becoming a chiropractor, practice characteristics
Clinical settings; urban/ rural, types of patients
**2. Study and research team factors**:
Reasons for choosing to participate in the study Thoughts on research and its relationship to chiropractors’ clinical practice
**3. Practitioner-related factors**:
Thoughts on current research project
Protocol, preparedness
Examples of when the recruitment process did not work out/worked out
Eligibility of patients, time constraints, economical constraints
**4. Patient factors**:
Experiences of patients’ interests, willingness to volunteer
**Reminders if not addressed during the general discussion**:
Challenges encountered, strategies to reduce such challenges
Collaboration between the chiropractors and research team, what was good, what could have been better (accessibility, information, communication)?
Dilemmas – and ways to overcome them

### Data analysis

Transcription of the interviews was performed using Autotekst, a digital service accessed within the secure TSD server. The transcripts were checked against the recordings for accuracy and anonymised. The data material included transcripts and fieldnotes. After reading the transcripts several times to gain an overall impression, a mismatch was noticed between the chiropractors’ expectations and motivations for participating in the trial and their actual recruitment experiences. This led us to direct further analysis to answer three analytical questions: What motivated clinicians to become co-researchers in the trial? What obstacles did they encounter when participating in the trial? How were these obstacles managed?

Transcripts were imported into NVivo 11, and relevant quotes were identified and coded. Two authors (AAU, AMM) coded the first interview independently, to enhance our reflexivity. AAU then coded the remaining four interviews. Themes were developed from ordering data by coding, grouping interrelated codes into categories [40], that were further clustered into themes, as shown in Table 2.

**Table 2:** Examples of the analysis - Theme development

Codes	Categories	Theme 1
For the greater good Bringing something to the table The importance of evidence Effects Side effects Negative attitudes of others	Professional commitment Participation to investigate the use of spinal manipulation	Participation as a co-researcher to contribute to professional development
**Codes**	**Categories**	**Theme 2**
Patients with acute neck pain were simply not there Not meeting the eligibility criteria Prior knowledge of the trial was important Not wanting to delay “real” treatment Time constraints Economic pressures Maintaining ones’ reputation	Recruitment was unexpectedly challenging Moral dilemmas	Recruitment was not as straightforward as expected and raised dilemmas
**Codes**	**Categories**	**Theme 3**
Trial promotion / Extra workload Teamwork Having enough time and energy Flexibility Only inviting new patients Referring to the protocol to justify recruitment	Obligations of multiple roles Protecting patient interests and themselves as breadwinners	Navigating conflicting obligations as a clinician, co-researcher, and breadwinner

Although one author conducted most of the coding (AAU), the transcripts and our interpretation were continuously discussed among the three authors with diverse scientific and professional backgrounds. The second author (NKV) is an experienced researcher with an extensive background in musculoskeletal disorders, physiology and quantitative research. The discussions continued during the writing process, which we view as an integral part of the analysis [41]. The analysis was inductive and grounded in the data, guided by how participants’ expressions were shaped by specific clinical contexts and group interactions. Quotes were translated into English by the bilingual first author (a native English speaker) to preserve meaning.

This study was reported in accordance with Consolidated Criteria for Reporting Qualitative Research (COREQ) checklist [42].

## Results

### Demographics and work contexts of the clinicians/co-researchers

Five men and 10 women aged 32–59 years with a clinical experience ranging from 6 to 34 years were interviewed. Most were Norwegians, with some being educated in the United Kingdom, others in Denmark, Australia, or the United States. Four practiced in rural and eleven in urban areas. Eleven worked in multidisciplinary clinics (e.g., with General Practitioners (GPs), physiotherapists, or osteopaths), and four worked with other chiropractors. The hectic lives of these chiropractors were expressed and mirrored by several; some were still at work and dressed in their clinic attire during the interviews scheduled at 7 p.m. Musculoskeletal complaints predominated the ailments treated; primarily NP or low back pain (LBP) among adults. Some also reported treating children and pregnant women. A few described more specialised services (e.g., diagnostic ultrasound, management of dizziness).

Our analysis is described under three themes (Table 2): (1) Participation as a co-researcher to contribute to professional development, (2) Recruitment was not as straightforward as expected and raised dilemmas, (3) Navigating conflicting obligations as a clinician, co-researcher, and breadwinner.

### Participation as a co-researcher to contribute to professional development

The chiropractors expressed a genuine commitment and motivation to take part in the trial. In general, they wished to contribute to professional development and to develop evidence to investigate the use of spinal manipulation for acute NP. The latter motivation was in response to public scepticism amid a current media debate about the risks of spinal manipulation of the neck. They expressed an awareness of the lack of high quality, clinically relevant evidence and viewed participation in a well-designed trial as necessary, given the emphasis on evidence-based practice (EBP) in PHC. This trial was therefore seen as “spot on”:

I feel that there is far too little research in this area [spinal manipulation of the neck]. And then I feel that a lot of PhD projects that chiropractors get are not about manipulation […] whereas this one project was very specific to what we do. So, I think that this study is very important for us as a profession, and what we do at the clinic every day. (Informant no. 005)

The chiropractors expressed hope that the trial would find spinal manipulation to be effective for acute NP, but some said that they would continue using the technique, even in the event of a negative result, since definitive conclusions cannot be drawn based on one study:

If it had come out that the manipulation did not work, then I think I would continue to manipulate. It was more to be able to show the world. (Informant no. 002)

The chiropractors agreed that it was important for their practice to overcome the scepticism regarding spinal manipulation held by other health care professionals, particularly physicians. Some of the chiropractors working in neighbouring clinics to GPs and other physicians, talked about receiving fewer referrals for acute NP, in contrast to acute LBP. They attributed this to physicians’ concerns of the perceived risks of spinal manipulation of the neck. They also expressed concern that such scepticism could influence patients’ attitudes toward their treatment:

I feel that there is a bit of fear among some patients. In relation to neck manipulation. In relation to the dangers. Many have heard something from a neighbour or GP, or maybe they have been to the emergency room. They may have received the comment that whatever you do, do not go to a chiropractor. If you’re going to see a chiropractor, don’t let them crack your neck. (Informant no. 001)

Considering public scepticism and a recent media debate about whether spinal manipulation of the neck increases stroke risk, the chiropractors saw the trial as timely for the evaluation of treatment effects and side effects. They hoped the results would support the safety of spinal manipulation, counteract recurrent scaremongering, demonstrate greater effectiveness of spinal manipulation than painkillers, and even influence national guidelines for acute NP.

### Recruitment was not as straightforward as expected and raised dilemmas

The chiropractors reported various recruitment experiences but were collectively surprised at how difficult it was. They had expected many patients with acute NP but found few, realising that their practices mostly saw patients with chronic or episodic NP or cases linked to specific specialisations (e.g., dizziness). Moreover, many patients with acute NP failed to meet the inclusion criteria (e.g., age, language barriers, comorbidities). A common surprise was that acute NP patients typically delayed seeking care for longer than those with acute LBP, often exceeded the trial’s definition of acute NP as an onset within the last 14 days, which clinicians linked to patients’ perceptions of NP as being less disabling than LBP:

I feel that people come more readily [to the chiropractic clinic] if they have acute LBP. Because they are too scared to walk and sit, while with acute NP pain, then you just have to turn the whole body, and it’s fine. (Informant no. 005)

Patients who learned about the study via newspapers or social media more often fulfilled the inclusion criteria and were typically more willing to participate than those without prior knowledge, who commonly declined participation. This was mainly because they expected spinal manipulation and feared delaying “real treatment” until after the trial’s 14-day intervention period. The clinicians also experienced that some patients had already tried painkillers without benefit or had an aversion to them, which further reduced their willingness to participate:

There are many people who read the [study] information and are enthusiastic. Then you get to the part about randomisation and that it is possible that they end up in a medicine group. Then they do not want to participate. (Informant no. 011)

At times, the chiropractors noted that some patients sought a “quick fix,” such as self‑employed fishermen needing a rapid recovery to work, or patients with employer-funded insurance who felt pressured to return quickly out of loyalty to their employers. Even when patients were eligible and willing, clinicians sometimes chose not to recruit them owing to their own time constraints, economic pressures, and concerns about disrupting established patient-clinician relationships. Although they knew that participation was unpaid, the unexpectedly long recruitment period created additional financial strain:

It is a bit difficult to talk about. When you mention it, if you start to calculate how much it may have cost [being a co-researcher], it is a lot of money. I knew that we were not going to get paid for taking part in the trial but in my opinion, I think that it may be a reason that some [of the recruiting chiropractors] do not recruit patients. (Informant no. 015)

The clinicians had to safeguard their practices as recruitment could affect their reputation in the community, something that was of particular concern among those with newly established clinics. On one hand, participating in research could be considered praiseworthy, “for the greater good,” and their participation without financial incentives showed that they took their professional work seriously. On the other hand, they expressed concern about their reputation being compromised, because dissatisfied participants receiving sham manipulation or medicines might not return or make recommendations to friends and family.

### Navigating conflicting obligations as a clinician, co-researcher, and breadwinner

The clinicians developed strategies for navigating multiple obligations when recruiting patients. They had been encouraged by the research group to promote the trial by targeting the media to gain attention. While the chiropractors recognised that this could reinforce their own reputation in society, such promotion was expressed by several as being time consuming, adding to the extra workload related to administrative tasks and screening patients. A suggestion to minimise this problem was that the research group should set aside extra resources for this purpose and that having a high‑profile academic spokesperson would be useful for larger scale promotion of the study.

The chiropractors generally used their clinics as a recruitment arena with help from their colleagues and held regular clinic meetings. Some made agreements that patients with acute NP presenting to the clinics would be directed to them:

I understand it [the project] requires a lot of time and effort to do, even though it is so important. But my colleagues are very eager to help me with the recruitment. (Informant number 015)

For some, recruitment was approached as a team and increased access to a greater number of eligible patients. Others felt uncomfortable with such arrangements because prolonged recruitment could reduce their colleagues’ income.

However, the most challenging part of recruitment was to reconcile competing obligations as clinician, co-researcher and breadwinner. When they had time, they contacted patients in advance to get an impression of their eligibility and streamline recruitment. But often, the clinicians had to assess eligibility and recruit spontaneously, which required them to have enough time, energy and impetus at that exact moment:

I think that time constraints are also a factor in this, with the fact that if you are a little late as it is and it is a current patient, it is very quick to… bet that there will be a new one next week instead. (Informant number 007)

Sometimes the protocol was prioritised by allotting extra time for recruitment. For example, more flexibility for recruitment was created by recruiting during lunch breaks, after ordinary working hours, or even at weekends. In doing so, the clinicians diminished disruptions for regular patients, but this was at the expense of their own free time:

I sat and ate lunch while he went about filling in the form. I would not have done that if it hadn’t been for the study. I usually try to be prepared before they arrive. (Informant no. 015)

Another dilemma raised was the delivery of sham manipulation over several consultations. Most clinicians found it morally problematic that sham manipulation could delay the patients’ recovery and felt that they were being dishonest:

I feel very false, that I am lying [to the patient] and that I am not giving the patient the help that they need. (Informant no. 015)

Thus, some clinicians did not recruit patients to avoid the issue completely. The clinicians also expressed that it became increasingly challenging to keep up the façade during five consecutive sessions, as the delivery of sham manipulation had a noticeably shorter duration than typical consultations. In contrast, other clinicians managed the discomfort by referring loyally to the research protocol. This transferred responsibility to the research group, enabling them to hide their discomfort more convincingly:

And when I am done [with sham manipulation] I always say… Now the protocol says we are done. I must treat according to the research protocol. (Informant no. 013)

Another strategy applied was to create their own inclusion criteria. This was not only as a means of protecting relationships with established patients, but also to protect their own reputations, and to prioritise their own interests as breadwinners. For example, some invited only new patients, naïve to spinal manipulation. New patients were considered easier to convince that sham manipulation was real compared to “experienced” patients. By contrast, dispensing pill boxes of medicines was perceived to be less problematic. This involved only a single visit; thus, it felt less awkward despite patients not receiving the treatment they were expecting:

I do not really talk much to those who get [allocated to the] medicine [groups]. They just get the pill box and go out the door. Thanks, and bye. (Informant no. 003)

When eligible patients declined to participate, the clinicians could do little. Attempts to convince patients were seldom successful, and over time they learned who was worth approaching. They developed strategies to balance duties as clinician, co‑researcher, and breadwinner as best as they could. However, the loyalties linked to being a co-researcher and clinician often conflicted. Many times, the patients’ interests came first, though this depended on the situation at that given moment. The clinicians believed they had exerted considerable effort to fulfil their role as co-researchers, yet they frequently felt caught up between competing obligations and limited time.

## Discussion

Our study shows that being a chiropractor and a co-researcher in this trial conducted in PHC was complex. This complexity arose not only from following the trial protocol, but from enacting it in a PHC setting, for example delivering sham treatments while maintaining a clinical reputation. A strong generic drive was to contribute to “a spot on” trial that could contribute to strengthening professional development and legitimise spinal manipulation of the neck. Yet, these intentions often collided with the contextual and relational realities of PHC, requiring ongoing negotiation between conflicting obligations, nested in the roles of clinician, co-researcher, and breadwinner.

### Legitimisation as a motivation to participate in the trial

The theme “Participation as a co-researcher to contribute to professional development” captures the interviewed chiropractors’ aspirations to contribute to strengthening the chiropractic profession and legitimise spinal manipulation of the neck through research. This aspiration to contribute to professional development is likely rooted in EBP, widely regarded as the basis for sound clinical practice [43–48], and a prerequisite for professional legitimacy [49]. A previous study showed that most Norwegian chiropractors value EBP [47]. Our findings support this: the interviewed chiropractors’ motivation to join the trial was grounded in how chiropractic is perceived in Norwegian PHC and the belief that higher‑quality research is needed to move chiropractic from the “sidelines” into a more recognised role in PHC. The strategy of strengthening a profession through research has also been important for other professions, such as physiotherapy [50].

The interviewed chiropractors’ more profession-specific aspiration concerned legitimising spinal manipulation of the neck, as an important part of their clinical practice. Their motivation seemed to be less about changing daily practice and more about supporting a central yet controversial treatment [51]. Still, a range of perspectives were found. Some stated that if spinal manipulation of the neck proved ineffective, changing practice would be necessary; others reasoned that clinical experience, and the limitations of a single study justified its continued use. Both views seem valid and illustrate the ongoing debate about whether evidence should inform or determine treatment choices [52].

### Moral dilemmas and their management

The theme “Recruitment was not as straightforward as expected and raised dilemmas” can be understood in terms of moral dilemmas and their management. Our study revealed two main areas of moral dilemmas; one was related to recruitment itself, and the other was related to the delivery of sham manipulation.

Recruitment challenges such as those in the current study include contextual-related factors (e.g. lack of eligible patients, reluctance to accept randomisation), practitioner-related factors (e.g. development of criteria to protect relationships with patients), and participant-related factors (e.g. patient preferences) that are common across clinical settings [7, 14, 18, 20]. Importantly, following a trial protocol often involves restricting treatment choices and negating treatment preferences. This can make it difficult for clinicians to meet patients’ expectations and address what is important to them. Our findings contribute to discussions in chiropractic about conducting research in routine practice, managing time, financial pressures, and handling ethical dilemmas [53–56]. Trials in other contexts, such as long-term and end-of-life care also have to contend with similar dilemmas related to randomisation, and the use of additional criteria to protect patients, but face other issues such as difficulties in obtaining consent, progressive deterioration, and even death [57–60]. These examples highlight the multitude of obstacles clinicians face when participating in clinical research. If clinical research and clinician involvement as co-researchers are to be optimised, such dilemmas must be addressed.

A central dilemma encountered in our study related to the delivery of sham manipulation. Many of the interviewed chiropractors found sham manipulation to be morally challenging. Challenges in delivering relational, manual sham interventions have been documented previously [61–63]. This is likely because spinal manipulation depends on a trusting social relationship between patient and clinician, unlike pill-based placebos, which are more straight-forward, as they typically involve minimal personal interactions [61], as noted by our interviewees. An additional difficulty was that clinicians could distinguish between active and sham interventions (in contrast to patients), requiring them to engage in active deception over follow-up visits, which intensified moral dilemmas. A key strategy to reduce this was to invite only new, naïve patients to the trial. This strategy of patient selection is reported to be common among clinicians partaking in trials, with the intent of protecting the interests of patients [7, 57, 58], but is problematic as it can introduce selection bias and threaten the representativeness of trials. In our study, this strategy also served to protect clinicians’ businesses and reputations, an aspect which is likely more specific to a PHC context where clinicians are self-employed. These findings underline moral dilemmas inherent to the delivery of relational sham interventions, with relevance for other professions applying similar modalities. From a research perspective, the moral and practical challenges of delivering sham/placebo treatment should be addressed to reduce recruitment bias but will depend on the research context [64].

### Context/structural aspects and the management of dilemmas

The context of trial recruitment is also an important consideration regarding the management of dilemmas. Most trials are conducted in hospital settings where recruitment and intervention delivery are shared among teams [17], and institutional infrastructures, employed positions, and collective responsibility buffer the potential consequences of patients’ trial experiences. In PHC, responsibilities for recruitment and trial intervention delivery, particularly relational types like spinal manipulation, fall largely on individual clinicians. Without institutional support/infrastructure, tensions and consequences (reputational and financial risk, loss of patients) are felt more directly. Individual clinicians acting as co-researchers have limited tools to mitigate dilemmas typical of clinical trial designs, and are left to devise their own solutions, as our study shows. These contextual aspects specific to PHC, help explain why protocol demands, including sham delivery, were experienced as especially burdensome and ethically difficult. They point to the need for institutions designing trials to assume greater responsibility for recruitment and participation, to offload the burden from individual clinicians.

### Negotiating roles through shifting loyalties to manage dilemmas

The theme “navigating conflicting obligations as a clinician, co-researcher, and breadwinner” can be understood as how the interviewed chiropractors negotiated multiple roles through shifting loyalties to manage dilemmas. Loyalty to the project group motivated them to “do their part” in the trial and contribute to the chiropractic evidence base. Yet, loyalty toward individual patients often took precedence. Many repeatedly emphasised their ethical duty to act in the patients’ best interests, consistent with previous literature [65, 66]. A third loyalty concerned their own livelihood: as self-employed clinicians, they worried that dissatisfied trial participants might not return, potentially harming their reputation and income. Adopting a co-researcher role was thus a complex, dynamic activity that had to be balanced with the clinician role in a highly relational, patient-specific PHC context. These roles entail differing interests and obligations that can generate ethical challenges [3, 8], making everyday practice a matter of moral decision-making [5, 11].

Our study presents a more complex account of PHC recruitment than earlier work rooted in realism; previous studies imply that challenges can be resolved by addressing individual practical barriers [12–14]. Using an interactionist perspective, we show how many of the interviewed chiropractors acted as moral agents, navigating shifting loyalties across the trial, the patient, and business/breadwinner axes. This broadens our understanding of the co‑researcher role in PHC, particularly when trials involve relational, manual sham interventions. To improve protocol adherence in PHC, researchers should acknowledge these interwoven context-dependent issues and seek to lessen their impact. We recommend large-scale trial promotion in the media etc. by researchers and/or their institutions, reduced administrative burden, reimbursement, and strengthened research support systems.

### Methodological considerations

A key strength of the current study is the research team’s diverse theoretical perspectives and professional backgrounds. This heterogeneity fostered critical questioning and continuous reflection throughout the research process, which helped us clarify, uncover and interpret nuances in the findings. We explicitly value this diversity and have strived to develop a transparent and coherent text to substantiate the trustworthiness of our study.

The interviewed chiropractors were all co-researchers involved in the same trial. Nonetheless, the interviews captured various experiences shaped by differing practice contexts which we consider important. Given this mix of common and different backgrounds, we consider the number of participating chiropractors to be adequate. It is unlikely that including more of them would have yielded substantially richer data. Involving chiropractors engaged in other trials, however, might have enriched the material.

A limitation is the small number of participants in several of the focus groups; one focus group consisted of only two participants, which may have constrained the discussions. However, our strategic sampling, bringing together chiropractors with no, limited, and extensive experience of recruiting to the trial, appeared to sustain a relatively wide range of perspectives, making areas of agreement, disagreement, and shared understanding more visible. In line with Malterud’s conceptualisation [67], we therefore consider our material to have sufficient information power to provide an adequate, although not exhaustive, account of the challenges of clinicians acting as co-researchers in PHC.

We argue that our findings are relevant for other clinicians in PHC. We focused on chiropractors participating as co-researchers in this trial and examined a contested intervention, spinal manipulation of the neck, and we identified challenges that are probably transferable to other PHC clinicians and their contexts.

## Conclusions

The clinicians’ motivations to take part in the trial varied but were in general rooted in a wish to contribute to strengthening the chiropractic profession. The negotiation of multiple roles and shifting loyalties rendered them moral agents. Our findings illustrate the social and relational nature of participating as a co-researcher in a trial conducted in a chiropractic PHC context. Our study also highlights a need for better support systems for research in PHC. This calls for political and organisational will to establish systems that provide necessary support to researchers and clinical co-researchers.

## Supplementary Information

Below is the link to the electronic supplementary material.

Supplementary Material 1

## Data Availability

The interview material is stored at the Services for Sensitive Data (TSD) at the University of Oslo and is not available to external parties in the interests of maintaining participant anonymity.

## Abbreviations

EBP: Evidence–based practice
GPs: General practitioners
LBP: Low back pain
NP: Neck pain
PHC: Primary health care
TSD: Services for Sensitive Data
